# Interleukin-27 Functional Duality Balances *Leishmania* Infectivity and Pathogenesis

**DOI:** 10.3389/fimmu.2020.01573

**Published:** 2020-08-07

**Authors:** Abdollah Jafarzadeh, Maryam Nemati, Prashant Chauhan, Ashok Patidar, Arup Sarkar, Iraj Sharifi, Bhaskar Saha

**Affiliations:** ^1^Department of Immunology, School of Medicine, Kerman University of Medical Sciences, Kerman, Iran; ^2^Immunology of Infectious Diseases Research Center, Research Institute of Basic Medical Sciences, Rafsanjan University of Medical Sciences, Rafsanjan, Iran; ^3^Department of Haematology and Laboratory Sciences, School of Para-Medicine, Kerman University of Medical Sciences, Kerman, Iran; ^4^National Centre for Cell Science, Pune, India; ^5^Trident Academy of Creative Technology, Bhubaneswar, India; ^6^Leishmaniasis Research Center, Kerman University of Medical Sciences, Kerman, Iran

**Keywords:** EBI3, IL-27, *Leishmania*, p28, WSX-1

## Abstract

IL-27 is a cytokine that exerts diverse effects on the cells of innate and adaptive immune systems. Chiefly expressed in macrophages and dendritic cells during the early phase of Leishmania infection, IL-27 contributes to the protection against L. major infection but suppresses the protective Th1 response against L. donovani, L. infantum, L. amazonensis and L. braziliensis infections, suggesting its functional duality. During the late stage of Leishmania infection, IL-27 limits the immunopathogenic reactions and tissue damages. Herein, we analyze the mechanism of the functional duality of IL-27 in the resistance or susceptibility to Leishmania infection, prompting IL-27 for anti-Leishmanial therapy.

## Introduction

**Figure 1:**
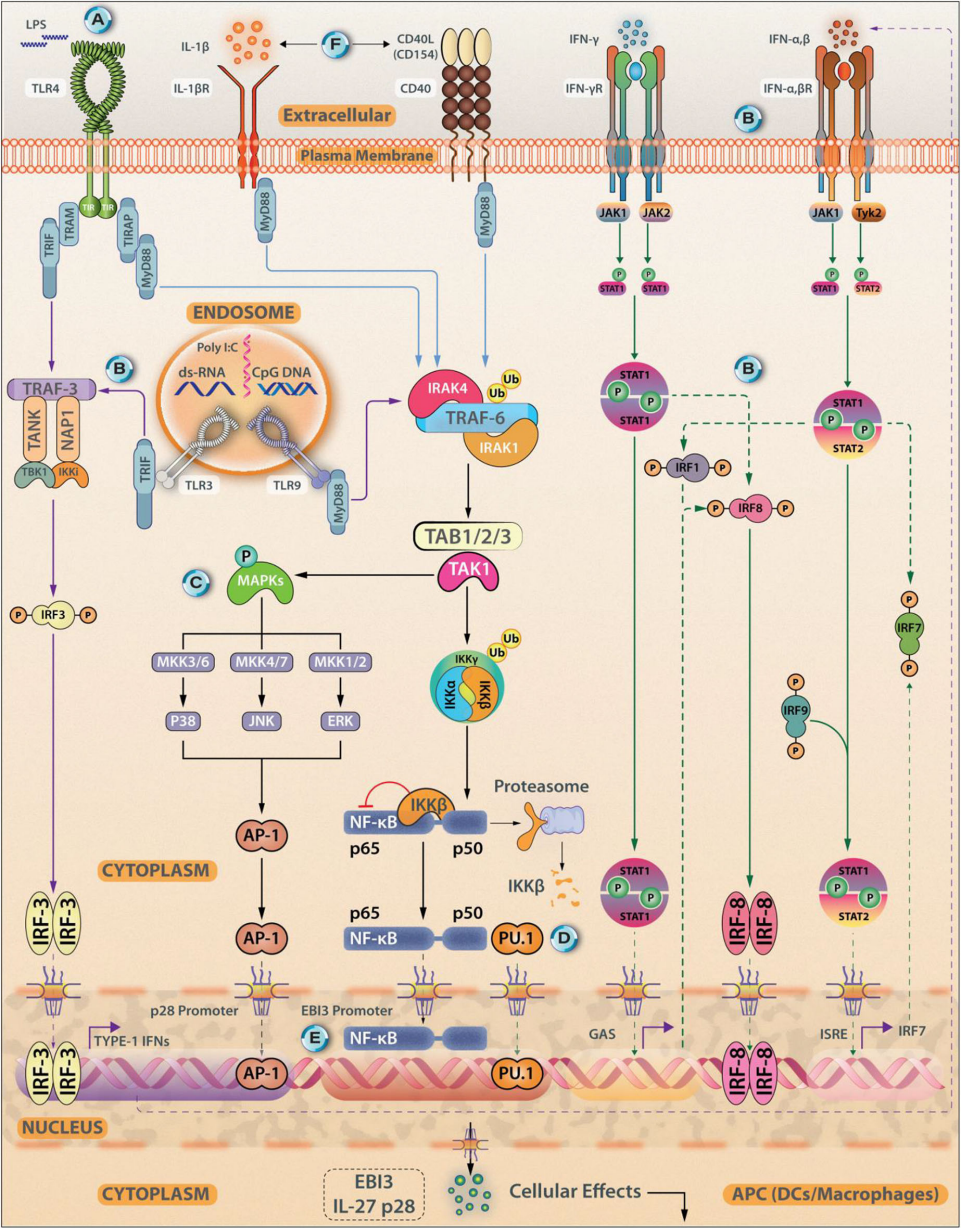
Transcriptional Control of IL-27 Synthesis. Antigen experienced immune cells (APCs, DCs, and macrophages) act as sources of IL-27. Various cellular receptors such as Toll-Like Receptors (TLR4), IL-1βR, and CD40 promote IL-27 synthesis. The expression of IL-27 subunits from APCs can be induced using TLR agonists Poly(I:C), lipopolysaccharide (LPS), and CpG-DNA, for TLR3, TLR4, and TLR9, respectively. **(A)** The expression of the IL27p28 subunit can be induced through signals coming from the TLR4/MyD88/AP-1/c-Fos signaling axis. **(B)** Other signaling axes that culminate in the induction of IL-27p28 synthesis are- TLR3/TLR4-TRIF-IRF3/IRF7; IFN-γ/MyD88/IRF8 and IFN-α/IFN-β-STAT1/STAT2-IRF1/IRF9. **(C)** IFN-γ can induce IL-27 expression *via* stimulating JNK, MAPKs, and PI3K signaling. **(D)** EBI3 expression is induced by signaling through TLR2/TLR4/TLR9-MyD88-NF-κB/PU.1. **(E)** Both p50 and p65 can bind to the EBI3 promoter. **(F)** Exogenous stimuli from IL-1βR or CD40-CD154 ligation can also trigger IL-27 synthesis in a MyD88 dependent fashion. Besides NF-κB, c-Rel, recruitment of PU.1, and IRF1 and IRF3 are essential events that regulate IL-27 synthesis by shaping its transcriptional landscape. Both IFN-γ, along with IFN-α and IFN-β are reported to be associated with p28 expression in DCs.

**Figure 2:**
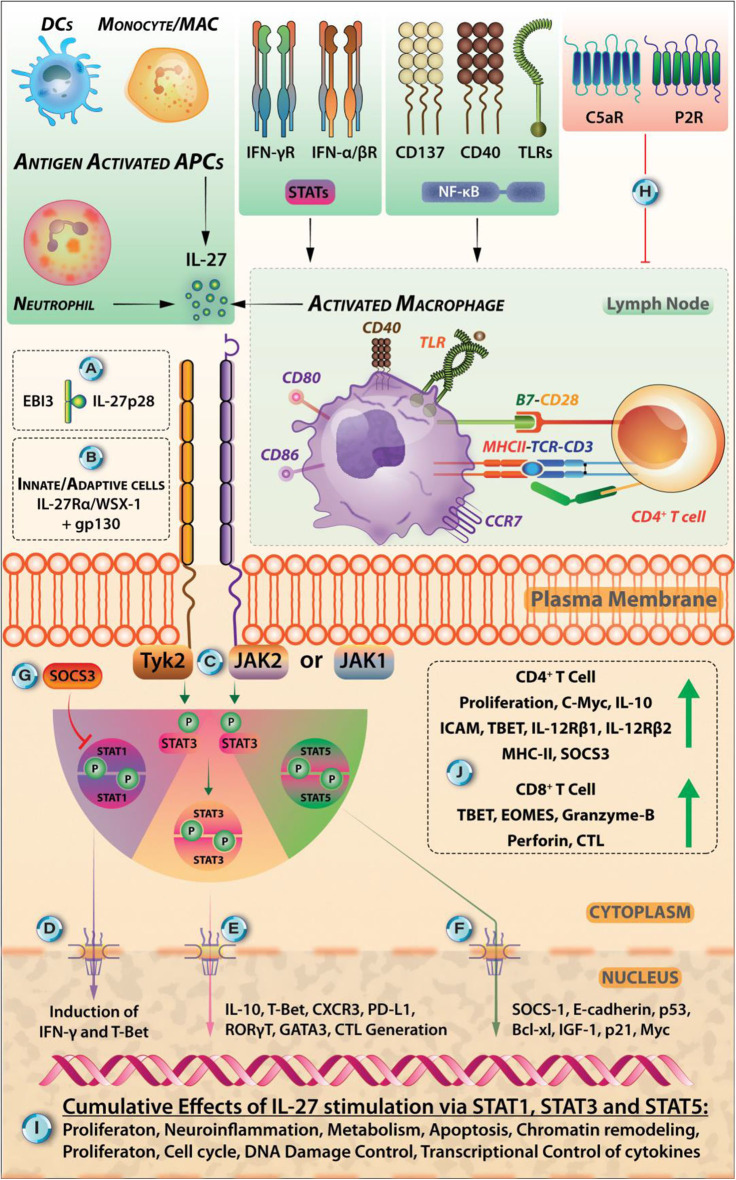
Various Extracellular Cues Can Induce IL-27 Signaling In STAT-1, STAT-3, AND STAT-5 Dependent Manner. **(A)** IL-27 is heterodimeric cytokine comprised of p28 and Epstein-Barr virus-induced gene 3 (EBI3) subunits. **(B)** IL-27 exerts its specific effects in immune cells through binding to its receptor IL-27R (comprised of WSX-1/IL27Rα and gp130). **(B)** IL-27R is expressed by cells belonging to both innate and adaptive modules of the immune system. **(C)** The intracellular signaling is initiated when IL-27 binds to its receptor, and this triggers the signal flow through kinases JAK1, JAK2, and Tyrosine kinase (Tyk2) that phosphorylates the subsequent isoforms of STAT proteins (mainly STAT-1, STAT-3, STAT-4, and STAT-5 each of which, has restricted expression in various immune cells) and promote their dimerization and subsequent nuclear translocation. **(D)** The nuclear translocation of STAT-1 dimers can lead to the induction of IFN-γ signaling and transcription factor T-bet that induces Th1 cells. **(E)** However, nuclear translocation of STAT3-dimers leads to the synthesis of IL-10, CXCR3, cytotoxic T lymphocyte induction, GATA-3, RORγt that may promote Th2, Th17 responses (not necessarily under the effects of IL-27). **(F)** Signaling through STAT-5—its dimerization, nuclear translocation, and DNA binding through tyrosine phosphorylation—can lead to the upregulation of SOCS-1, E-cadherin, p53, Bcl-xL, p21, and Myc proteins. **(G)** The signals through IL-27R are carefully regulated in the cell *via* its intrinsic regulation through SOCS-1 and SOCS-3, maintaining a negative feedback loop. **(H)** The extracellular cues coming from extracellular ATP binding to purinergic receptors (P2R) in the case of inflammation and C5a binding to C5aR are antagonistic signals for IL-27 synthesis. **(I)** Broad effects of IL-27 relating to proliferation, in regulating cell cycle, neuroinflammation, metabolism, apoptosis, chromatin remodeling, and transcriptional control are mediated by STAT-1, STAT-3, and STAT-5 signaling pathways. **(J)** IL-27 stimulation in CD4^+^ T cells leads to their proliferation, expression of c-Myc, IL-10, ICAM, T-bet, IL-12Rβ1, IL-12Rβ2, MHC-II, and SOCS3. It also promotes the STAT1-, STAT3-dependent generation of IL-10 secreting Tr1 cells. IL-27 promotes CD8^+^ CTL generation, causes upregulation of T-bet, Eomesodermin (EOMES), and Granzyme-B. IL-27-induced modulation of host-pathogen relationships is an area described in this review in the context of protozoan parasite *Leishmania*.

IL-27 is a heterodimer of p28 and Epstein-Barr virus-induced gene 3 (EBI3) subunits and signals through IL-27-receptor complex, a heterodimer of IL-27Rα WSX-1 and gp130 subunits (1). The major producers of IL-27 are activated antigen-presenting cells (APCs), including DCs and macrophages (2, 3). Signaling axes, including TLR4-MyD88-AP1/c-Fos, TLR4-MyD88-IRF1/IRF8-NF-κB-c-Rel, TLR3/TLR4-TRIF-IRF3/IRF7, IFN-γ-MyD88-IRF8, and IFN-α/IFN-β-STAT-1/STAT-2-IRF1/IRF9 induce the p28 expression (4–7). However, p38MAPK overexpression activates c-Fos that inhibits p28 expression (2, 8). By stimulating the C-Jun N-terminal kinases (JNK), MAPKs, and the phosphoinositide-3-kinase (PI3K), signaling IFN-γ induces IL-27 expression in human monocytes (5). The EBI3 expression is induced by the signaling axes TLR2/TLR4/TLR9-MyD88-NF-κB/PU.1 (9– 11) (Figure 1). Although IL-27 is secreted as p28-EBI3 heterodimers, the IL-27p28 subunit can be secreted without EBI3. p28 (or IL-30) may work as IL-27 antagonist while working through IL-27Rα (12) but may work as an agonist through IL-6 receptor; the mechanism behind these contrasting effects remains unclear (13). The IL-27Rα exhibits the cellular and tissue/organ specificity for IL-27 effector functions. Various cells including activated B cells, activated endothelial cells, DCs, macrophages, mast cells, monocytes, naïve T cells, NK cells, express IL-27R (12, 14, 15). The diversity of IL-27R-mediated intracellular functions depends on the activation of different isoforms of JAK and STAT present in the immune cell subsets: For instance, JAK-1, JAK-2, Tyrosine kinase-2 (TYK-2), STAT-1,−2,−3,−4, and−5 in naïve CD4^+^ T cells, JAK-1, STAT-1, STAT-3, and STAT-5 in NK cells, STAT-1 and STAT-3 in monocytes, and STAT-3 in mast cells (12). The binding of IL-27 to its receptor initiates the JAK/STAT signaling pathway (Figure 2), which involves phosphorylation of mainly STAT-1, STAT-3, and STAT-5 transcription factors (TFs) (16). The induction of SOCS3 (17), however, inhibits IL-27 signaling through a negative feedback loop, by inhibiting JAK-activity (18). By virtue of all these immunomodulatory functions, IL-27 plays significant roles in Leishmaniases, a complex of diseases inflicted by the protozoan parasite of the genus *Leishmania*, as described below.

## IL-27 Functions in Innate and Adaptive Immune Systems

Upon stimulation with TLR ligands, IFNs and CD40-ligand, DCs, and macrophages release IL-27 that work in an autocrine and paracrine manner to mount innate and adaptive immune responses in several diseases; by contrast, many pathogens induce the synthesis of IL-27 in these cells.

### Effects of IL-27 on Innate Immunity

IL-27 promotes NK cell cytotoxicity through upregulation of perforin and granzyme B expression (2) and supports the IFN-γ production by inducing T-bet, a transcription factor, and by enhancing the IL-18 responsiveness (19). IL-27 treatment of mast cells and eosinophils enhances their adhesion and survival and induces the production of pro-inflammatory cytokines such as IL-1, TNF-α, and IL-6 (20). IL-27 limits neutrophil recruitment and reduces IL-6 and IL-12 secretion from these cells (21). IL-27 enhances the differentiation of monocytes into macrophages, stimulates macrophages to produce nitric oxide, and induces moDCs to express IL-27, IL-8, CXCL10, chemokine receptor (CCR1), IRF8, and IFN-stimulated genes (3). IL-27-pretreated monocytes show activated STAT-3 and NF-κB phosphorylation, elevated TLR4 expression, and with LPS co-treatment, increase IL-6, TNF-α, MIP-1α, and MIP-1β expression (22). By contrast, IL-27 induces the expression of the immunosuppressor indoleamine 2,3-dioxygenase (IDO) in humans monocytes (18, 23).

**Figure 3:**
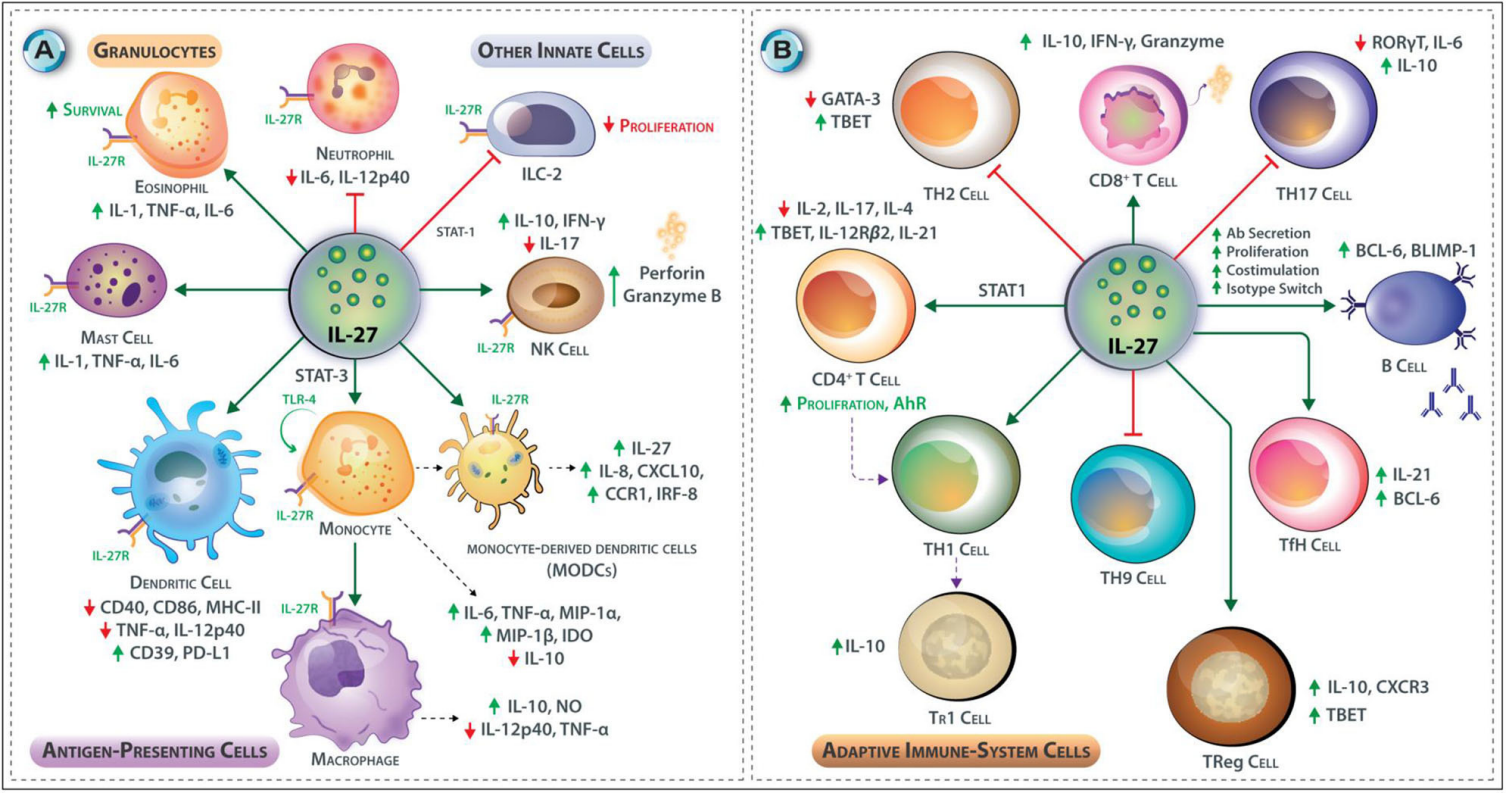
**(A,B)** Effects of IL-27 on Innate and Adaptive immune responses. **(A)** IL-27 promotes cytotoxicity in NK cells through upregulation of perforin and granzyme B, and it induces IFN-γ production from NK cells *via* T-bet transcription factor but inhibits IL-17 production in NK cells. In mast cells and eosinophils, IL-27 promotes pro-inflammatory cytokine synthesis and release; these include IL-1, TNF-α, IL-6, promotes adhesion and survival in eosinophils. Contrary, IL-27 limits neutrophil recruitment and reduces the secretion of IL-6 and IL-12p40 from these cells. IL-27 enhances TLR4 expression by monocytes through STAT-3 and NF-κB and enhances their differentiation to macrophages. In macrophages, it induces NO expression and triggers moDCs to express IL-27, IL-8, CXCL10, CCR1 IRF8, and IFN-stimulated genes. IL-27 also induces the expression of an immunosuppressive enzyme IDO in human monocytes. IL-27 inhibits DC functions; stimulation of DCs with IL-27 before LPS reduces expression of CD40, CD86, and MHC-II but upregulation of CD39 and PD-L1. IL-27 may also inhibit the secretion of TNF-α from DCs. IL-27 inhibits the innate lymphoid cells (a subgroup of innate cells that lacks specific antigenic receptors) proliferation through STAT-1. **(B)** IL-27 induces T-bet and IL-12Rβ2 expression in CD4^+^ T cells at the early phase of T cell polarization. IL-27 inhibits the production of IL-2 by CD4^+^ T helper cells through induction of SOCS-3. IL-27 promotes IL-10-producing Tr1 cells. IL-27 inhibits the development of Th2 cells by downregulating GATA-3. IL-27 interferes with Th17 development by suppression of RORγt and interfering with IL-6 signaling it also stimulates IL-10 production and induction of PD-L1 on naïve T cells. IL-27 inhibits the development of Th9 cells. IL-27 supports the development of Tr1 CD4^+^ T cells. IL-27 also promotes the development of IL-10 secreting T-bet^+^ CXCR3^+^ Treg cells. T follicular helper (Tfh) cells produce IL-21 in the presence of IL-27. CD8^+^ T cells are also affected by the presence of IL-27 as it increases their proliferation and capacity to secrete IFN-γ, and granzyme B. IL-27 regulates many aspects of B cell development and their functions. In response to IL-27, B cells increase expression of Bcl-6 and Blimp1, which is critical for plasma cell differentiation.

IL-27 inhibits DC functions, as WSX-1-deficient DCs are hyper-reactive to LPS and promote NK cells and T cells to produce higher amounts of IFN-γ than that induced by wild-type DCs (24). IL-27 pre-treatment of DCs reduces the LPS-stimulated expression of the MHC-II and costimulatory molecules—CD40, CD86—perhaps due to IL-27-induced elevated CD39 expression (24, 25). B7-H1 (PD-L1), which provides suppressive signals to T cells, is upregulated by IL-27 in DCs (10, 26, 27). Consistent with these observations, IL-27 reduces HLA-restricted antigen presentation and inhibits proliferation and cytokine production of allogeneic T cells (28). Through STAT-1 activation (29), IL-27 inhibits proliferation and cytokine production by type-2 Innate lymphoid cells (ILC2)-a specialized cell-type that lacks antigen-specific receptors but produces high levels of helper T-cell cytokines and lipid mediators in response to antigen-independent stimuli. ILC2 express GATA3 and partake in airway inflammation *via* the production of type-2 cytokines. A summary of the effects of IL-27 on innate immune cells is shown in Figure 3A.

### Effects of IL-27 on the Adaptive Immune System

Naïve CD4^+^ T cells abundantly express IL-27Rα or WSX-1, but IL-2 activated and differentiated CD4^+^ T cells show reduced IL-27Rα expression (30). IL-27 induces T-bet and IL-12Rβ2 expression in CD4^+^ T cells imparting IL-12 responsiveness during the early phases of Th1 polarization (Figure 2) (31). By contrast, IL-27 inhibits IL-2 production from CD4^+^ Th cells through SOCS3 induction, limiting T cell responses, and as IL-2 is responsible for the proliferation and survival of Th1 cells, IL-27 mediates suppression of Th1 cell-related immunity (32). Suppression of Th1 cell response by IL-27 also can be explained by the induction of IL-10 an anti-inflammatory cytokine (33). IL-27 inhibits Th2 cell development and production of Th2 cytokines in already polarized Th2 cells by downregulation of GATA3 expression and upregulation of T-bet expression (34). IL-6 and TGF-β trigger naïve CD4^+^ T cells differentiation to Th17 cells by inducing RORγt expression (35). IL-27 inhibits the Th17 cell differentiation through interference with IL-6 signaling and suppression of the expression RORγt, induction of the IL-10 production and autocrine IL-10-mediated inhibition, and induction of programmed death-ligand 1 (PD-L1) on naïve T cells (36, 37).

Two subsets of Treg cells are generated from precursor cells in the thymus including natural Treg (nTreg) and inducible Treg (iTreg) cells, or antigen-induced naive CD4^+^ T cells in the presence of TGF-β and IL-2, in the peripheral tissues, respectively. Treg cells express the transcription factor FoxP3 and are characterized by the secretion of immunosuppressive cytokines TGF-β, IL-10, and IL-35 (38). The iTReg were divided into two subgroups, including Tr1- and Th3-cells, which are characterized by the production of the large amounts of IL-10 and TGF-β, respectively (39). Indeed, IL-27 supports the development of immune-regulatory Tr1 CD4^+^ T cells (40). IL-27 also promotes the development of IL-10, producing T-bet^+^ CXCR3^+^ Treg cells (41). IL-4 and TGF-β elicit naïve CD4^+^ T cells differentiation to Th9 cells, which express the transcription factor PU.1 and secrete large amounts of IL-9 that contribute to mast cell activation, induction of allergic reactions, and immune responses to intestinal helminth infections (42). IL-27 inhibits the development of Th9 cells (43). IL-6 and IL-21 elicit the T follicular helper (Tfh) cell differentiation from naïve CD4^+^ T cells. Tfh cells express Bcl6—a transcription factor—and provide CD40L, ICOS, and IL-21 signals required for B cell proliferation and differentiation (44). IL-27 promotes Tfh cell polarization and induces IL-21 (45), which promotes B cell expression of Bcl-6 and Blimp-1 that are critical for plasma cell differentiation and B cell function. Although IL-27 induces B cell proliferation and antibody production, it does not seem to promote the formation of memory B cells (46).

CD8^+^ T cells recognize viral- and tumor-derived antigenic peptides presented by MHC-I molecules and play a fundamental role in the killing of virally infected and cancer cells. IL-27 enhances the proliferation of naïve CD8^+^ T cells and the production of IFN-γ and granzyme B (47) (Figure 2). IL-27 thus differentially affects the functions of immune cells in both innate and adaptive immune systems. The effects of IL-27 on adaptive immune cells is summarized in Figure 3B.

## The Role of IL-27 in *Leishmania* Infection

*Leishmania* is a protozoan trypanosomatid parasite and is transmitted by the sand fly vectors. *Leishmania* exists as extracellular, flagellated motile promastigotes in sand flies and as sessile, aflagellate, amastigotes within the mammalian macrophages in skin, liver, spleen, bone marrow, and mucosal tissues. It causes a complex of diseases called Leishmaniases to affect 12 million people worldwide and one billion at risk (48, 49). Depending on the invading specie of *Leishmania*, tissue tropism of the parasite, the site and the route of infection and pathophysiology, Leishmaniases are classified into cutaneous (CL), mucocutaneous (MCL), diffuse cutaneous (DCL), visceral (VL), and post-kala-azar dermal Leishmaniasis (PKDL) (49). The CL is the most prevalent form of the disease (70–75%), caused by *L. major* and *L. tropica* in the Middle East and North Africa, while *L. mexicana* and *L. braziliensis* are predominant in South America (49, 50). *L. amazonensis* causes DCL and CL with disseminated lesions. *L. donovani* and *L. infantum* cause VL that disseminates to spleen, liver, and bone marrow (51). Thus, *Leishmania* species differ in their growth kinetics, the tissue of infection, and clinical outcomes. Although macrophages serve as the host for all these *Leishmania sp*. the immunopathology of *Leishmania* is greatly affected by tissue-specific non-immune cells. IL-27 plays a complex role in the immuno-pathology of Leishmaniasis (52).

### Do *Leishmania* Salivary Gland Proteins (SGPs) Affect IL-27 Secretion?

Most species of *Leishmania* are transferred through the phlebotomine sand-fly vector. The metacyclic promastigotes are embedded in proteophosphoglycan-rich promastigote secretory gel (PSG) secreted by themselves in the anterior midgut of sandflies. PSG must be regurgitated along with infective promastigotes before sand flies can begin hematophagy on host skin. In turn, this event leads to the accumulation of PSG, *Leishmania* along with sand-fly saliva containing SGPs on host skin. Since the first description of sand-fly SGPs disease-exacerbating function in CL (53), these are shown to induce cytokines and chemokines with potential contrasting functions (54). Many proteins including LJM19, a novel 11-kDa protein, are identified as anti-Leishmanial vaccine candidates (55, 56). Although IL-27-inducing SGPs are not reported, some probable pathways may be proposed. First, as the sand-fly bite is rich in immunomodulatory components like CD39 family ecto-apyrases converting ATP and ADP to AMP and Pi (57), the adenosine receptors on the infected DC and macrophages may modulate IL-27 secretion (58). Second, as platelet activation is associated with the chemotactic migration of effector monocytes, a potent source of IL-27, to the sites of *L. major* infection (59) an axis of SGPs-platelets activation-monocyte recruitment may modulate IL-27 production affecting the outcome of *Leishmania* infection. Third, salivary-homogenates from *Lutzomiya longipalpis* suppress the costimulatory molecule expression and CD40L-induced DCs maturation (60), which may lower available IL-27 pools as observed with IL-10 and TNF-α. A limitation with such studies is that these do not highlight the exact specific composition of sand-fly salivary gland extracts, which may be composed of more than 35 secreted proteins (61). Fourth, although a vasoactive peptide (maxadilan) from the *L. longipalpis* saliva is shown to indeed increased production of prostaglandin E2 (PGE_2_) (62) and reciprocally modulate TNF-α and IL-6 production from BALB/c macrophages in a PGE_2_-dependent manner (62), the regulation of IL-27 by maxadilan and a correlation between PGE_2_ and IL-27 in experimental Leishmaniasis require further investigation. Similarly, SP15-like protein, D7-related proteins, lufaxin (reversible inhibitor of factor Xa of coagulation cascade), palmitoyl-hydrolase, and YRP proteins (57) may modulate the host IL-27 production by previously unexplored mechanisms. More research is needed to confirm the definitive roles of salivary gland homogenates in immunosuppression and interception of the IL-27 secretion axis in the case of *Leishmania sp*. infection.

### The Role of IL-27 in *L. major* Infection

**Figure 4:**
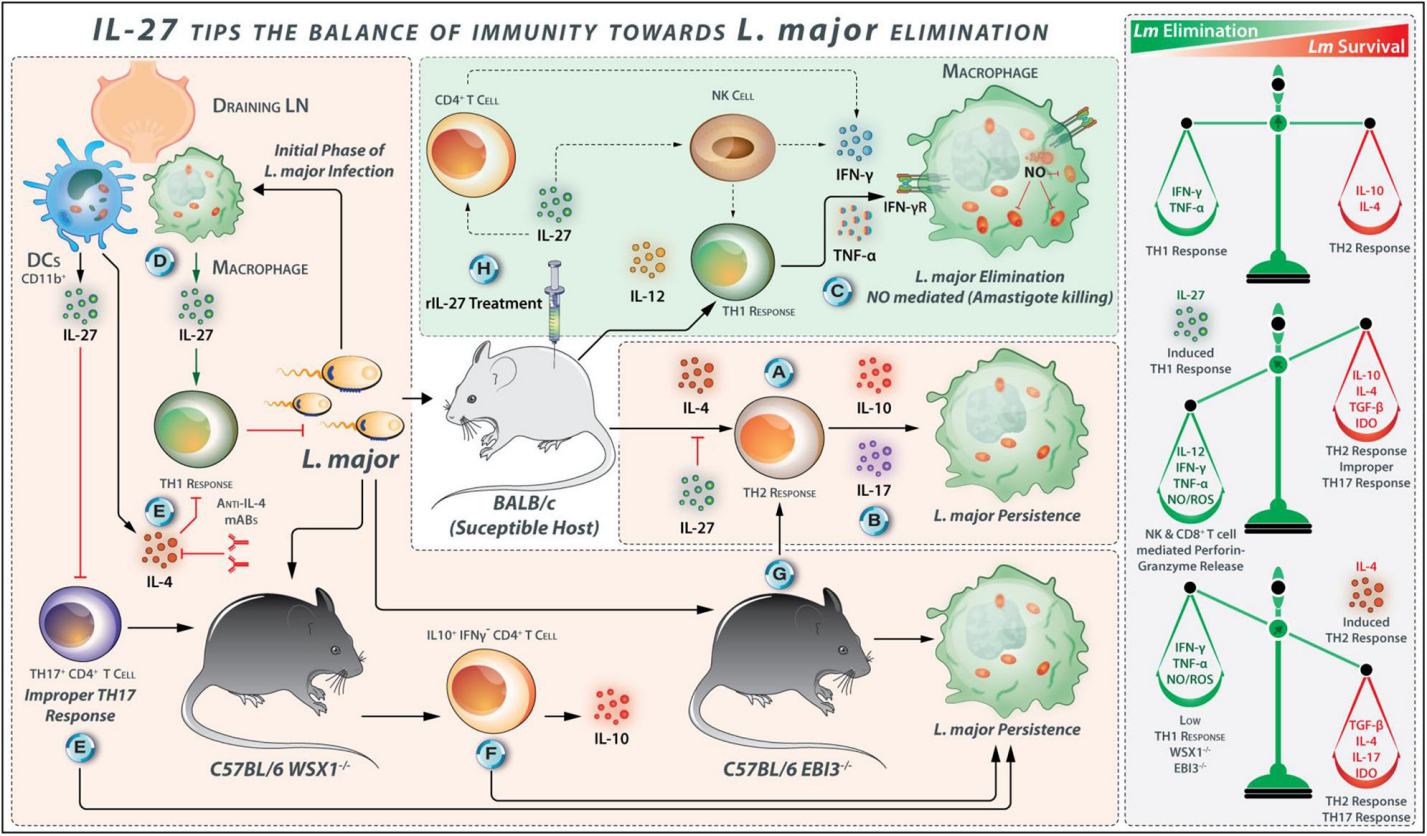
IL-27 Tips the Balance of Immunity Toward L. major Elimination. **(A)** *Promotion of L. major growth by Th2 response-* IL-10 and IL-4 are identified as a factor for susceptibility in BLAB/c mice because these cytokines are drivers of Th2 type response, which promotes *L. major* growth. **(B)** Repression of IL-10 signaling increases IL-17 production and exacerbates *L. major* infection. **(C)** However, TNF-α and IFN-γ cumulatively induce iNOS expression in *L. major*-infected macrophages for parasite clearance. **(D)** IL-27 is identified as a factor that promotes resistance to *L. major* infection in mice. *L. major*-infected CD11b^+^ DCs are major sources of IL-27 secretion in the draining lymph nodes of C57BL/6. **(E)** In WSX1^−/−^ mice, the initial immune response is accompanied by secretion of IL-4 by *L. major*-infected cells, and IL-4 neutralization during the early phase of infection may abolish the requirement of IL-27 for the development of an effective Th1 response. The role of IL-27 in the prevention of improper IL-17 cell development is also mentioned. **(F)** In WSX1^−/−^ mice, the low percentage of IL-10^+^ IFN-γ^−^ CD4^+^ T cells—associated with the development of more severe lesions—are found. **(G)** Similarly, in EBI3^−/−^ mice, the Th1/Th2 balance is diverted toward Th2 response indicating the protective roles that IL-27 plays for immunity against *L. major*. **(H)** IL-27 stimulates CD4^+^ T cells and NK cells to secrete IFN-γ during the early stages of *L. major* infection. Therefore, exogenous delivery of IL-27 into *L. major*-infected BALB/c mice exhibits protection by direct suppression of Th2 response and induces protective Th1 response.

*L. major* infection results in polarization of the Th cells to either IL-12-dependent host-protective IFN-γ secreting Th1 cells or IL-4-dependent disease-promoting IL-10 and IL-4 secreting Th2 cells in resistant or susceptible mouse strains, respectively (63). The *L. major*-infected BALB/c mice display high IL-17 levels, and IL-17 deficiency or IL-17 neutralization results in a reinforced host-protective anti-Leishmanial response (64). Likewise, IL-17 quantities are positively associated with skin inflammation in patients with CL and MCL (65). IFN-γ and TNF-α synergistically induce the expression of inducible nitric oxide synthase (iNOS) that catalyzes nitric oxide production in macrophages to eliminate *Leishmania* (65, 66). The iNOS-deficient mice are vulnerable to *L. major* infection despite the presence of robust Th1 cell response, indicating an essential role of NO in limiting *Leishmania* infection (63, 67). Adding the effects of IL-27 in these complex inter-regulated circuits develops a better understanding of the CL. High levels of IL-27 expression are observed in *L. major* infection *in vivo*. The p28 expression increases at the second week, whereas EBI3 expression becomes measurable in the fifth week in the *L. major-*infected C57BL/6 mice (68). CD11c^+^ DCs express both IL-27 subunits, including p28 and EBI3, in the draining lymph nodes of *L. major*-infected C57BL/6 mice at 24 h post-infection (69) (Figure 4). Therefore, the infected DCs are the major producers of IL-27 during the early phase of CL.

### IL-27 Treatment Suppresses Th2 Responses and Protects the Host From *L. major* Infection

The IL-27 signaling *via* WSX-1 contributes to protection against *L. major* infection (69–71). Upon IL-27 treatment, IL-27 transgenic mice are protected against *L. major* infection (34). The daily IL-27 administration during the first seven days after *L. major* infection of BALB/c mice reduces the parasite load and increases the survival rate compared with control mice (34). The lymph node cells from *L. major*-infected IL-27-treated BALB/c mice secrete higher quantities of IFN-γ and produce lower amounts of IL-4 in comparison with the cells from control mice (34). Therefore, the exogenous IL-27 protects *L. major*-infected BALB/c mice from CL by direct suppression of the Th2 cell responses accompanied by the induction of host-protective Th1 cell responses, perhaps due to its ability to augment IFN-γ production from NK and CD4^+^ T cells (70, 71). The *L. major*-infected macrophages from patients with healing forms of lesion produce a higher amount of IL-27 and IL-23 as compared with patients having non-healing forms of the lesion, suggesting that IL-27 and IL-23 may play synergistic roles with Th1 cytokines in protection against Leishmaniasis (72). *L. major* lysate-stimulated lymphocytes from EBI3^−/−^ mice secrete fewer IFN-γ and high IL-4, IL-10, and IL-13 as compared to the lymphocytes from the control mice on the second and third week post-infection. Thus, at early time points after infection, the Th1/Th2 cell balance in EBI3^−/−^ mice is diverted toward Th2 cells (69).

#### IL-27 Deficiency Impairs Host-Protective Th1 Responses and Induces Susceptibility to *L. major* Infection

In WSX-1^−/−^ mice both IFN-γ production and resistance to *L. major* are impaired (69, 71). EBI3^−/−^ C57BL/6 mice also display greater susceptibility to *L. major* infection compared to C57BL/6 wild-type mice, with the maximum differences in the parasites loads and lesion sizes at the sixth-week post-infection; the lesions and parasite load are reduced from the eighth week, and lesions are entirely resolved till 14th week in EBI3^−/−^ mice (69). Rechallenge of healed EBI3^−/−^ mice with *L. major* does not lead to the elicitation of the pathological symptoms, indicating that the expansion and persistence of the protective immunologic memory are not impaired in EBI3^−/−^ mice (69). These observations suggest the importance of EBI3 for early control of parasite replication (Figure 4). Similarly, when compared with wild-type mice, the lymph node cells from WSX-1^−/−^ mice produce fewer IFN-γ initially but makes up the deficiency in the later stages of the infection. However, both WSX-1^−/−^ and wild-type mice successfully control the parasite and resolve the cutaneous lesions (71). Susceptibility to *L. major* infection in WSX-1^−/−^ mice is restricted to the initial stages of infection when a considerable level of IL-4 is produced (71). The anti-IL-4 mAb administration during the early stages of infection improves the IFN-γ-dependent host resistance in *Leishmania* infected WSX-1^−/−^ mice. The treatment of the WSX-1^−/−^ mice with blocking anti-IL-4 antibody also reduces *the T. cruzi* parasite burden and also increases the number of IL-17^+^ CD4^+^ cells, indicating that IL-4 inhibits the Th17 cell development in parasitic infections (73, 74). The initial IL-4 production during *L. major* infection determines the necessity for IL-27 in the induction of Th1 cell responses to limit the CL (Figure 4). However, IL-27 is not required to maintain an extended Th1 response during the later stages of *L. major* infection. Although WSX-1^−/−^ mice show significant lesions at an early phase of infection, at later stages of infection it ultimately limit parasite loads and resolve the infection (70, 71). Accordingly, the increased susceptibility of WSX-1^−/−^ mice to *L. major* is accompanied by the *Leishmania*-induced IL-4 production initially; therefore, blockade of IL-4 during the initial phase of infection abolishes the early requirement for IL-27 for the development of effective anti-parasitic responses. At 6–8 weeks post-infection, IFN-γ production is increased, and the IFN-γ/IL-4 ratio shifts toward Th1 cells in WSX-1^−/−^ and wild type mice, which were accompanied by parasite killing and lesion resolution (69). The early EBI3 expression is vital for the quick generation of effective Th1 cell-mediated immunity, but healing and immunologic memory develop in an IL-27-independent manner (69).

#### IL-27 Contributes to Early Development and Maintenance of Th1 Cells and Mediates Immunity Against *L. major*

It is proposed that IL-27 is initially produced by the parasite-stimulated phagocytes and skews Th1 response before IL-12 production is upregulated (75). Indeed, the IL-27-stimulated T cells express IL-12R and are then differentiated to Th1 cells in the presence of IL-12 *in vivo* (34). Early production of IL-27 may result in Th1 cell differentiation, and later on, maintained by IL-12 produced from *L. major-*infected DCs (76). Therefore, *L. major*-infected DCs sequentially produce IL-27 and IL-12 to establish and maintain protective Th1 cell-mediated protective immunity against this pathogen. Delayed IL-12 production during *L. major* infection may cause a delay in the initiation of Th1 responses (77). Indeed, WSX-1 expression diminishes in differentiated Th1 cells; contrastingly, IL-12R is still abundantly expressed in activated Th1 cells. Th1 cells from WSX1^−/−^ mice display normal IFN-γ production in response to IL-12. Therefore, IL-27 is necessary for the early development of Th1 cells, and IL-12 is necessary for early differentiation, establishment, and full activation of Th1 cells. IL-27 causes Th1 cell differentiation through the induction of p38MAPK that upregulates T-bet. Moreover, it promotes the expression of ICAM-1/LFA-1 adhesion molecules to induce ERK1/2-related signaling pathways. Induction of both signaling pathways causes IL-12Rβ2 expression, which promotes the Th1 differentiation and IFN-γ production in the presence of IL-12 (2). WSX-1-related signaling also induces STAT-1-dependent expression of T-bet and trigger IFN-γ production (78), and WSX-1^−/−^ mice display defects in IFN-γ expression (71). IL-27R-deficient mice exhibit enhanced Th2 cell-dependent asthmatic manifestations (79). Consequently, IL-27 determines the suppression of Th2 cell response. IL-27 inhibits Th2 cell differentiation and associated cytokine-production from fully activated Th2 cells (12). IL-27 also directly inhibits GATA-3 expression in a STAT-1-dependent pathway (80). IL-27 reduces GATA-3 expression while inducing T-bet expression even in polarized Th2 cells (34).

#### Regulatory Effects of IL-27 During *L. major* Infection

Severe lesions are reported in *L. major*-infected WSX-1^−/−^ mice, which were associated with the appearance of the IL-17^+^ CD4^+^ cells, indicating a role for IL-27 in preventing the improper development of Th17 cells during Leishmaniasis (68). The IL-17-deficient BALB/c mice exhibit smaller lesions during *L. major* infection, although a modest reduction in their parasite load is observed (64). The addition of rIL-27 to *L. major* lysate-stimulated CD4^+^ T cells collected at the eighth week post-infection from *L. major-*infected C57BL/6 mice enhances the population of IL-10^+^ IFN-γ^+^ CD4^+^ cells and IL-10^+^ IFN-γ^−^ CD4^+^ cells. As compared with the wild-type C57BL/6 mice, WSX-1^−/−^ C57BL/6 mice display a low percentage of IL-10^+^ IFN-γ^−^ CD4^+^ cells and more severe lesions 6 weeks after *L. major* infection (68). Therefore, the development of more severe lesions in the *L. major*-infected WSX-1^−/−^ mice may partly be attributed to the downregulation of the anti-inflammatory cytokine IL-10. IL-27 does not interfere with IFN-γ, TNF-α, or IL-17 production by the soluble *Leishmania* antigens (SLA)-stimulated PBMCs from patients with CL and MCL. PBMC cultures from patients with CL and MCL when supplemented with IL-27 do not show any change in the production of IL-10 (81). These findings indicate that the cytokine-producing cells from patients with CL and MCL are unresponsive to the regulatory effects of IL-27.

#### IL-27 Is Dispensable for Maintaining Extended Protective Th1 Cell Response Against *L. major*

IL-27 expressed by the infected-phagocytes and DCs during the early phase of *L. major* infection contribute to the protection during the initial stage of *L. major* infection, while considerable levels of IL-4 are present and IL-4-induced Th2 cell responses are dominant (82). The protective effects of IL-27 may be exerted through inhibition of IL-4 production, prevention of Th2 cell response (34), and induction of the IFN-γ production from CD4^+^ T and NK cells, which reinforces Th1 cell responses. However, IL-27 is not necessary to maintain an extended protective Th1 cell-related response during the late stage of *L. major* infection (70). IL-27 limits tissue damages and immunopathology during *L. major* infection *via* preventing the improper Th17 cell responses (68) and induction of the immunoregulatory cytokine IL-10. Based on the discussion above, the regulatory effects of IL-27 during *L. major* infection are compiled and summarized in Figure 4.

### The Role of IL-27 in *L. donovani* Infection

**Figure 5:**
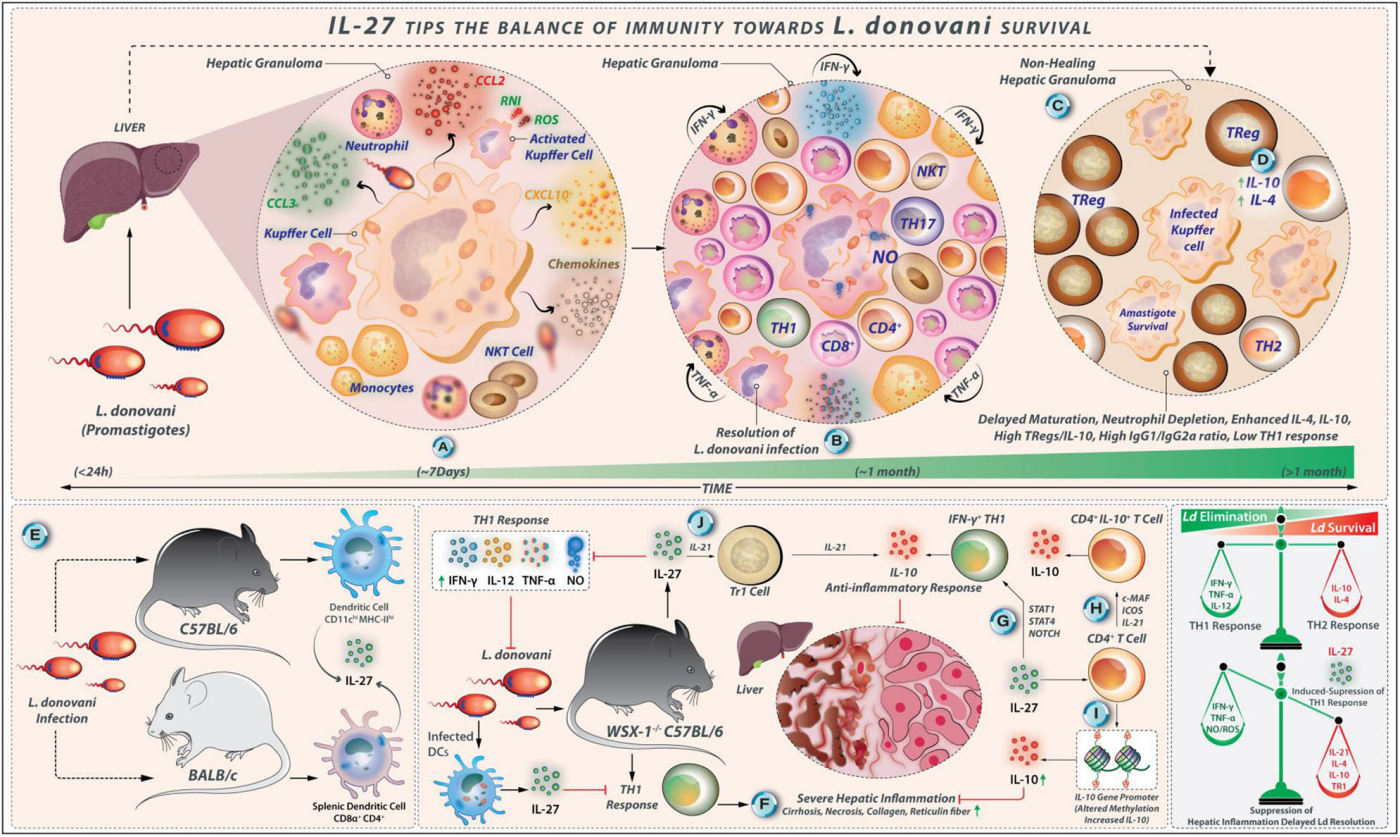
IL-27 tips the balance of immunity toward *L. donovani* survival. **(A)** *L. donovani* amastigotes invade hepatic macrophages (Kupffer cells). This event is followed by the invasion of leukocytes, neutrophils, monocytes, and T cells under the influence of chemotactic factors like CCL3, CCL2, and CXCL3 secreted by infected Kupffer cells. **(B)** These immunological reactions lead to the formation of a granuloma that is abundant in pro-inflammatory factors (IFN-γ, TNF-α, and RNI/ROS secreted by Th1 cells, NK cells, and CD8^+^ T cells); under such conditions *L. donovani* infection is resolved over time. **(C)** However, the abundance of IL-4 and IL-10 decreased iNOS expression, high production of IgG1/IgG2a ratio, and delayed maturation of granuloma, depletion of neutrophils results in high parasite load. **(D)** Elevated IL-10 secreted by Treg cells also contributes to *L. donovani* survival. **(E)** Splenic CD11c^hi^ MHC-II^hi^ DCs from *L. donovani*-infected C57BL/6 mice show high levels of IL-27. Similarly, *L. donovani* infection in BALB/c mice produces IL-27. In this case, CD8α^+^ and CD4^+^ DCs are the major producers. **(F)** In WSX-1^−/−^ C57BL/6 mice, a potent Th1 cell-linked response exhibits a severe hepatic inflammation (showing necrosis and deposition of reticulin fiber and collagen) that eventually resolves. In this context, IL-27-linked signals may suppress the Th1 response directly or indirectly, which helps in the suppression of hepatic inflammation by modulating the function of CD4^+^ T cells. **(G)** IL-27 supports the IL-10 production by IFN-γ producing Th1 cells in a pathway that involves the induction of STAT-1, STAT-3, and NOTCH signaling. **(H)** The generation of CD4^+^ IL-10^+^ T cells by IL-27 requires the expression of c-Maf, ICOS, and IL-21. **(I)** IL-27 also alters methylation patterns in the IL-10-gene promoter in CD4^+^ T cells that increase IL-10 expression directly. **(J)** IL-27, along with IL-21, enhances the differentiation of IL-10 producing TR1 cells during VL.

A large number of the *L. donovani*-infected persons are asymptomatic because they develop an effective T-cell-mediated immune response against the parasite (51). Accordingly, the prevalence of visceral Leishmaniasis (VL) is low, and recuperated patients are mainly protected against reinfection. Several genetic parameters, helminth infections, and malnutrition are associated with susceptibility to VL (63). *L. donovani* amastigotes initially infect the liver macrophages, namely as Kupffer cells (Figure 5). Then the leukocytes, especially neutrophils, monocytes, and T cells infiltrate into the liver and form granulomas that are essential in controlling the hepatic infection. The Th1 cell-related cytokines, particularly IFN-γ and TNF-α, induce the ROS and reactive nitrogen intermediates (RNI) in Kupffer cells that contribute to the parasite elimination (Figure 5). *L. donovani* also infects the macrophages of spleen and bone marrow (51). Splenic chronic infection is associated with modification in the lymphoid compartments, apoptosis in T cells, and unresponsiveness to parasite antigens. The TNF-α-deficient mice and anti-TNF-α antibody-treated mice did not exhibit the changes in the marginal zone macrophages after *L. donovani* infection, demonstrating the essential role that TNF-α plays an in the alterations of the spleen compartments (83). *L. donovani* also stimulates splenic regulatory DCs to secrete great amounts of IL-10 after *in-vitro* stimulation (84).

#### IL-27-Linked Signals May Influence the Infection Outcome Against *L. donovani* by Direct or Indirect Suppression of the Protective Th1 Cell-Mediated Immune Responses

The depletion of neutrophils during the initial stages of *L. donovani* infection results in the high parasite load in the bone marrow and spleen, leading to splenomegaly, delayed maturation of hepatic granulomas, and a decrease the iNOS expression within the granulomas. Along with enhanced IL-10 and IL-4 quantities (85), high IgG1/IgG2a ratio against *L. donovani*, and elevated IL-10 production by Treg cells, the susceptibility to VL is associated with the inability to induce active cell-mediated immunity against *L. donovani* (86). The IL-10 neutralization promotes IFN-γ and TNF-α production and decreases the parasite load (87). *L. donovani* infection is not well-established in IL-10-defective mice, and the blocking of IL-10-related signaling enhances anti-parasitic immunity (87). *L. donovani* induces the differentiation of Th17 cells, which secrete cytokines, such as IL-17 and IL-22. The *L. donovani*-derived antigens stimulate PBMCs from healthy individuals to produce IL-17 and IL-22, *in vitro* (88). The Th1 and Th17 cells may perform complementary roles in protecting against *L. donovani* infection so that the impairment in the Th17 cell functions causes susceptibility to VL (88, 89). The splenic CD11c^hi^ MHC-II^hi^ DCs collected on day 21 and day 28 from *L. donovani*-infected C57BL/6 mice express high IL-27 levels (Figure 5). The *L. donovani* infection in BALB/c mice promotes IL-27 expression by splenic CD8α^+^ and CD4^+^ DCs on days 1, 14, and 28 post-infection (90). These results revealed that different DC subsets might be the primary IL-27 secreting cells during *L. donovani* infection. The circulating IL-27 quantities are elevated in *L. donovani*-infected humans, and splenic myeloid cells act as major producers of IL-27 (91). Indeed, the WSX-1^−/−^ C57BL/6 mice show resistance to *L. donovani* infection; in addition, it effectively controls parasite load compared to wild-type mice (92). The WSX-1^−/−^ C57BL/6 mice generate a potent Th1 cell-linked response that limits parasite growth; however, these *L. donovani*-infected mice display severe hepatic inflammation that eventually resolves. IL-27-linked signals may indirectly or directly suppress the protective Th1 cell-mediated immune responses against *L. donovani* infection (Figure 5).

#### IL-27 Deficiency Correlates With Robust Th1 Cell Response After *L. donovani* Infection

It was observed that the liver and spleen from *L. donovani*-infected WSX-1^−/−^ C57BL/6 mice show lower parasite loads compared with the wild-type mice on days 15 and 30 after infection (92). By day 60 after infection, both WSX-1^+/+^ and WSX-1^−/−^ mice efficiently control *L. donovani* loads in their liver and spleen. However, parasite loads in the liver are significantly lower in WSX-1^−/−^ mice (92). Therefore, IL-27 is dispensable for the development of immunity against *L. donovani* but delays the resolution of the parasite infection. Apart from this, the *L. donovani*-infected WSX-1^−/−^ mice display higher serum levels of IFN-γ and TNF-α on day 15 post-infection, more elevated serum IL-12 levels on day 30 post-infection compared with WSX-1^+/+^ mice (92). Levels of IL-12, TNF-α, and IFN-γ decline in *L. donovani*-infected WSX-1^−/−^ mice by day 60. Nonetheless, the *L. donovani*-infected WSX-1^+/+^ mice produce more IL-10 at all examined time points when compared with WSX-1^−/−^ mice, but differences were not significant (92). The *Leishmania* antigen-stimulated spleen cells from *L. donovani*-infected WSX-1^−/−^ mice produce more amounts of IFN-γ and IL-12 on day 30 post-infection and generate greater levels of NO on days 15 and 30 after infection in comparison to WSX-1^+/+^ mice. The splenocytes from WSX-1^−/−^ mice also produce more IL-10, indicating that IL-10 may be involved in the limiting of splenic but not hepatic inflammation in WSX-1^−/−^ mice following *L. donovani* infection (92). IL-4 is not produced by *L. donovani* antigen-stimulated spleen cells from both mouse strains at all-time points examined (92). These findings demonstrate that WSX-1^−/−^ mice establish a strong Th1 cell response after *L. donovani* infection.

#### IL-27 Plays a Critical Role in Suppressing Hepatic Inflammation but Does Not Impart Protection Against *L. donovani* Infection

Contrary to *L. major* infection, IL-27-related signaling is not required for the induction of the protective Th1 cell responses during *L. donovani* infection. There are some differences between *L. donovani* and *L. major* regarding the clinical, pathological, and immunological aspects. *L. major* infection in humans usually causes self-resolving cutaneous lesions, whereas *L. donovani* infection is related to systemic manifestations of parasite spreading in organs such as the liver, spleen, and bone marrow (63) (Figure 5). Although Th1 polarization is key to the protection from *L. major* infection, the protection against *L. donovani*-mediated visceral Leishmaniasis is associated with mixed Th1- and Th2-type cytokine responses; as a result, the cured VL patients display a mixed Th response (93). Although IL-4 is a vulnerability factor in mouse *L. major* infection, it is necessary for efficient vaccine-induced protection against *L. donovani* infection (93). WSX-1^−/−^ mice display severe liver pathology after *L. donovani* infection. WSX-1^−/−^ mice exhibit severe liver pathology with large granulomas associated with diffuse foci of inflammation and necrosis, as well as more deposition of collagen and reticulin fiber on days 15 and 30 after infection compared with wild-type mice (92). Therefore, IL-27 plays a critical role in suppressing hepatic inflammation during the acute phase of VL. As mentioned, *L. donovani*-infected WSX-1^−/−^ mice produce greater levels of IL-12, IFN-γ, and TNF-α during the early phase of infection in comparison to wild-type mice. The depletion of CD4^+^ T cells or neutralization of both TNF-α and IFN-γ also reduces the severity of liver pathology in WSX-1^−/−^ mice but renders them susceptible to *L. donovani* (92). Hence, the IL-27-mediated pathways suppress the tissue inflammation associated with VL by modulating the CD4^+^ T-cell function (Figure 5). However, blockade of IFN-γ or TNF-α alone is not sufficient to block liver inflammation or compromise host resistance in WSX-1^−/−^ mice (92). Therefore, IFN-γ or TNF-α production alone may not be adequate to induce immunopathology in the liver. These findings demonstrate that the IL-27-related pathways, in contrast to its protective role in *L. major* infection, are involved in the pathogenesis of *L. donovani* infection.

#### Immunomodulatory Effects of IL-27 During L. Donovani Infection

It has been indicated that the IL-10^+^ IL-27^+^ DCs can promote IL-10 production by Th1 cells *in vivo* (94). In *L. donovani* infected C57BL/6 mice, the frequency of splenic IFN-γ^+^ IL-10^+^ T-bet^+^ CD4^+^ T cells is increased on day 28 post-infection (94). IL-27 directly alters methylation patterns in CD4^+^ T cells at the IL-10 gene promoter, thus causing higher IL-10 expression (95) (Figure 5). IL-27 supports IL-10 production by IFN-γ-producing Th1 cells through STAT1, STAT4, and Notch signaling molecules *via* an alternate pathway (96, 97). The optimal generation of CD4^+^IL-10^+^ T cells by IL-27 requires c-Maf, ICOS, and IL-21 expression (98, 99). Further, IL-27 and IL-21 together enhance the expansion and differentiation of IL-10-secreting Tr1 cells during VL (40, 51). IL-21 also reinforces the IL-10 secretion by IL-27-induced Tr1 cells (100). Therefore, IL-27 controls the severity of VL-associated inflammation by inducing anti-inflammatory cytokine IL-10. Recently, in a mouse model of *L. donovani* infection using BALB/c mice, it has been reported that the administration of the neutralizing anti-EBI3 antibody (but not anti-p28 antibody) to pathogen-infected mice reduce the parasite load in the spleen and liver, and increase the TNF-α- and IFN-γ-secreting cells (101). As EBI3 is shared by IL-35 and IL-27, it has been concluded that IL-35 may have more immunomodulatory effects during *L. donovani* infection (101). These regulatory effects of IL-27 during *L. donovani* infection are summarized in Figure 5.

Collectively, IL-27 is expressed by different DC subsets during the early phase of *L. donovani* infection, IL-27 may directly and/or indirectly suppress the protective Th1 cell-mediated responses against *L. donovani* infection, which may delay the resolution of the parasite infection. The *L. donovani*-infected WSX-1^−/−^ mice exhibit a diversion in Th1/Th2 cells toward Th1 cell responses. During *L. donovani* infection, CD4^+^ T cells play a critical role in mediating hepatic damage in WSX-1^−/−^ mice wherein IL-27 attenuates immune-pathological response-related tissue damages by inducing the expression of the anti-inflammatory cytokine IL-10 by Tr1- and Th1 cells.

### Role of IL-27 in *L. infantum* Infection

*L. infantum* infection in humans and animal models leads to an increase in the IL-27 expression (102). High levels of the p28 subunit of IL-27 are detected in the spleen and liver of *L. infantum*-infected C57BL/6 mice at 4 and 6 weeks post-infection. The expression of both IL-27R subunits, including WSX-1 and gp130, are upregulated mainly in the fourth week after infection, indicating that the expression of IL-27 and its receptor is induced during *L. infantum* infection (103). The *in-vitro* experiments indicated that the bone-marrow-derived macrophages (BMDMs) and BMDCs from C57BL/6 mice can produce IL-27 when infected with *L. infantum* (103). The *Leishmania*-derived nucleic acids (DNA and RNA) are recognized by intracellular TLRs like TLR3, TLR7, TLR8, and TLR9 (63, 104). The BMDMs are deficient in TLR3 and TLR9 or adaptor molecules MyD88 and TRIF are not able to produce IL-27 after *L. infantum* infection, indicating that nucleic acids from *L. infantum* activate these pathways to trigger the IL-27 production. *L. infantum* infection induces IL-27 production in a consecutive process that includes the events, engagement of TLR3 and TLR9, the IFN-β induction, and activation of IRF1 that lead to IL-27 production in macrophages infected with *L. infantum*. The soluble CD40L also increases the production of several cytokines (IL-12p70, IL-23, IL-27, IL-15, and IL-1β) by *L. infantum-*infected human macrophages *in vitro* (105). A negative correlation was observed between the levels of these cytokines in the supernatants of cultured macrophages with the number of infected macrophages as well as amastigotes. *Leishmania* may also trigger sCD14 and initiate the IL-27, IL-10, and IL-6 production, which subsequently modulate macrophage microbicidal activity, facilitating *Leishmania* proliferation (106).

#### IL-27 Is a Regulatory Cytokine That Dictates Susceptibility to *L. infantum* Infection

For experimental VL both C57BL/6 and BALB/c strains are considered as susceptible models, but only BALB/c mice, not C57BL/6 mice displayed the augmented serum IL-27 levels during an early stage of *L. infantum* infection. The phenomenon was attributed to the upregulation of p28 expression by splenic DCs and higher parasite burdens in BALB/c mice (102). High expression of TLR2 by DCs from BALB/c mice may support the early IL-27 expression after infection, which contributes to the attenuating of inflammation and promoting infection (102). It has been reported that the *L. infantum-*infected BMDCs from BALB/c mice produce higher IL-27 levels than C57BL/6 mice. Whereas, the LPS-stimulated BMDCs from C57BL/6 mice produce higher amounts of IL-27 than BALB/c mice (102), indicating that parasite vigorously upregulates the secretion of IL-27 in BMDCs derived from BALB/c, but not from C57BL/6 mice. In both cutaneous and visceral forms of human Leishmaniasis, the IL-27 production is increased when the disease is in the active phase (91, 106). When compared with healthy subjects and delayed-type hypersensitivity (DTH) positive individuals, the sera from VL patients before treatment showed high levels of cytokines- IFN-γ, IL-10, IL-6, IL-27, and TNF-α. Besides, the serum levels of these cytokines decrease after treatment (106). B cell activation is also promoted by IL-27, exacerbating the hypergammaglobulinemia in VL patients (106). In a mouse model of VL, IL-27 plays a role in the suppression of the protective immune response during *L. infantum* infection, which can lead to disease exacerbation (103). IL-27 suppresses the immune response in VL through the induction of IL-10 production *in vivo* (91). The IL-27 restricts the Th1 cell polarization *via* inducing the IL-10-producing DCs. In fact, in the p28^−/−^ mouse model restricted to DCs, CD4^+^ T cell-related IFN-γ response is exacerbated (107). The collective production of both IL-27 and IL-10 by *L. donovani*-infected DCs is essential for IL-10 production by Th1 cells, resulting in parasite persistence (94).

#### IL-27 Modulates IL-17 to Affect Neutrophil Infiltration in the Spleen of *L. infantum* Infected Mice

**Figure 6:**
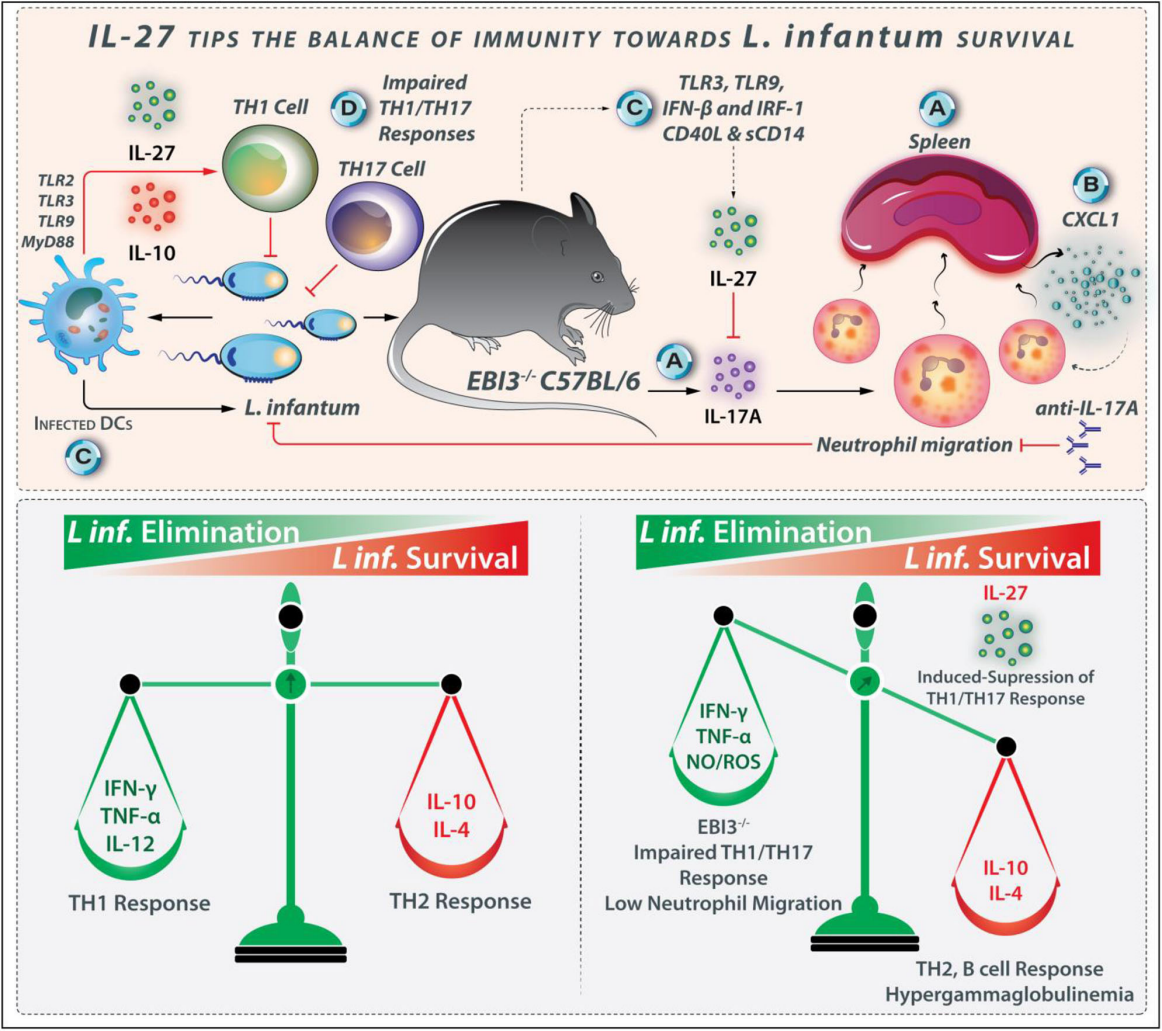
IL-27 tips the balance of immunity toward *L. infantum* survival. IL-27 plays a role in the suppression of the protective immune response during *L. infantum* infection. **(A)** In EBI^−/−^ mice, IL-27 modulates the IL-17 production and neutrophil infiltration. **(B)** Higher expression of powerful neutrophil chemoattractant CXCL1 is observed in the spleen of EBI3^−/−^ mice. **(C)** IL-27 and its receptor expressed during the early phase of *L. infantum* infection where infected DCs and macrophages are the major producers of this cytokine in pathways originating from TLR3, TLR9, and IFN-β. **(D)** As Th1 and Th17 cells-related responses help combat *L. infantum* infection, IL-27 is regarded as the susceptibility factor because of its ability to interfere with Th1- and Th17-induced protection.

The administration of rIL-27 to in C57BL/6 increases the IL-10 production, while decreasing IFN-γ and IL-12p70 in the peritoneal cavity, and prevents the infiltration of neutrophils in the spleen 24 h after the treatment (102). IL-27 neutralization in acutely infected BALB/c mice reduces parasite burdens and reduces IL-10 levels, while leads to a transient increase in the splenic IFN-γ-producing CD4^+^ and CD8^+^ T cells (102). The investigations using EBI3^−/−^ mice indicate a role for IL-27 in modulating the IL-17 production and neutrophil infiltration during the chronic phase of *L. infantum* infection (102, 103). The EBI3^−/−^ mice produce considerably higher IL-17A levels than wild-type C57BL/6 mice in both spleen and liver. Restimulation of EBI3^−/−^ mice-derived splenocyte with *L. infantum* lysate leads to significant IL-17A production. IL-17A is the critical mediator of neutrophil infiltration in the spleen of infected mice (Figure 6), which induces chemoattractant-CXCL1 expression (103). Higher CXCL1 expression is observed in the spleen of EBI3^−/−^ mice, which causes elevated neutrophil migration at the fourth week post-infection in the spleen and liver.

#### IL-27 Leads to Host Vulnerability to *L. infantum* by Reducing the Neutrophil Influx

Neutrophils have diverse mechanisms for combating pathogens, some of which include the production of cytokines and chemokines that lead to inflammatory cell recruitment as well as the release of proteolytic-enzymes/cathepsin/neutrophil-elastase and neutrophil extracellular traps (NETs) mediated-pathogen killing (108, 109). Host resistance through neutrophils in response to *Leishmania* depends on both the host's genetic background as well as the invading *Leishmania* species (63). For example, *L. amazonensis* promastigotes are eliminated by NETs, while *L*. *major* may survive within neutrophils (110). The host's genetic factors also affect neutrophil performance during Leishmaniasis. The neutrophil-defective C57BL/6 mice display a common *L. major* infection, while neutrophil-defective BALB/c mice exhibit a weak harmful Th2 cell response (111). The neutrophil-defective Genista mice are resistant to *L. mexicana* that causes non-healing lesions. During VL, neutrophils play a protective role. In C57BL/6 mice during *L. infantum* infection, IL-17A levels are reduced in the infected organs (103). The downregulation of IL-17A is mediated by IL-27, as EBI3 deficiency leads to intensified IL-17A secretion. Upon IL-17A neutralization, the resistant EBI3^−/−^ mice become as susceptible as the control wild-type mice, representing the ability of IL-27 to prevent the IL-17A production directly. Moreover, neutrophil infiltration is also reduced after anti-IL-17A treatment, satisfying the role of IL-17A in neutrophil recruitment to infected organs in parallel to the induction of NO production by macrophages (103). The inhibition of IL-17A by IL-27 during *L. infantum* infection leads to host vulnerability by reducing the neutrophil influx. Although the EBI3-deficiency impairs the Th1 responses in *L. infantum*-infected mice, other arms of the inflammatory response such as IL-17A and neutrophils limit the parasite load even in the absence of efficient Th1 cell response (103). Based on the discussion above, the regulatory effects of IL-27 during *L. infantum* infection are shown in Figure 6.

IL-27 and its receptor are expressed on the infected macrophages and DCs during the early phase of *L. infantum* infection. TLR3- and TLR9-, and IFN-β related signaling pathways play a prominent role in IL-27 production during *L. infantum* infection (Figure 6). In addition to the mouse strains, other molecules such as soluble CD40L and sCD14 also influence IL-27 production (112). In *L. infantum*-infected humans with VL, the IL-27 levels are increased in the active phase, and coming back to basal amounts after treatment, represent that this cytokine may be considered as a marker of VL severity or prognosis. IL-27 is a regulatory cytokine that plays a role in susceptibility to *L. infantum* infection. As Th1- and Th17 cell-related responses confer protection against *L. infantum* infection (113), IL-27 increases the susceptibility to parasite by interfering with both protective Th1 and Th17 responses.

### Role of IL-27 in *L. amazonensis* Infection

The C57BL/6 mice are vulnerable to *L. mexicana* or *L. amazonensis* infection due to compromised Th1 cell responses (114). This inability has been attributed to elevated IL-10 production, which causes insufficient DC activation and IL-12 production. Although Th1 cell-mediated immunity limits the *Leishmania* infection, the Th1 cell-released cytokines like TNF-α and IFN-γ involve in the immunopathologic reactions. Indeed, elevated TNF-α and IFN-γ quantities were detected in severe CL and ML patients. *L. amazonensis* causes anergic DCL and CL with disseminated lesions (50). A different modulating role has been proposed for Treg cells in *L. amazonensis* infection. The Treg transfers from *Leishmania*-infected mice to naive mice shortly before *L. amazonensis* infection decreases lesion development signifying that the role of Treg, cells in limiting immunopathological responses (115). The results from an *in-vitro* analyses indicate that the expression of both IL-27 subunits, including p28 and EBI3, is augmented in *L. amazonensis*-infected macrophages from C57BL/6 mice measured at 4 hours after infection (116).

#### IL-27 Is a Susceptibility Factor During *L. amazonensis* Infection

In *L. amazonensis*-infected C57BL/6 mice, the injection of the rIL-27 into the infected footpads (on days 2, 4, and 6 prior infections) strongly enhances the lesion sizes and parasite number in the footpads and the draining lymph nodes at weeks 2 and 3 following infection (116). Although IL-27 signaling contributes to the Th1 cell-related protective response in the *L. major*-infected mice, the same pathway may enhance the susceptibility to *L. amazonensis*, perhaps due to the differences in protein kinase R (PKR)-mediated signaling between *L. major*- and *L. amazonensis*-infected macrophages. The PKR-mediated signaling supports *L. amazonensis* but reduces *L. major* infection (117). *L. amazonensis* activates PKR in macrophages for their survival. The PKR activation by poly I:C (a ligand for TLR3) causes IL-10 production, and IL-10 neutralization prevents the parasite expansion (118), suggesting a TLR3-PKR-IL-27-IL-10 axis in *L. amazonensis* infection.

#### TLR2-PKR-IFN-1 Signaling Contributes to *L. amazonensis* Survival in an IL-27 Dependent Manner

**Figure 7:**
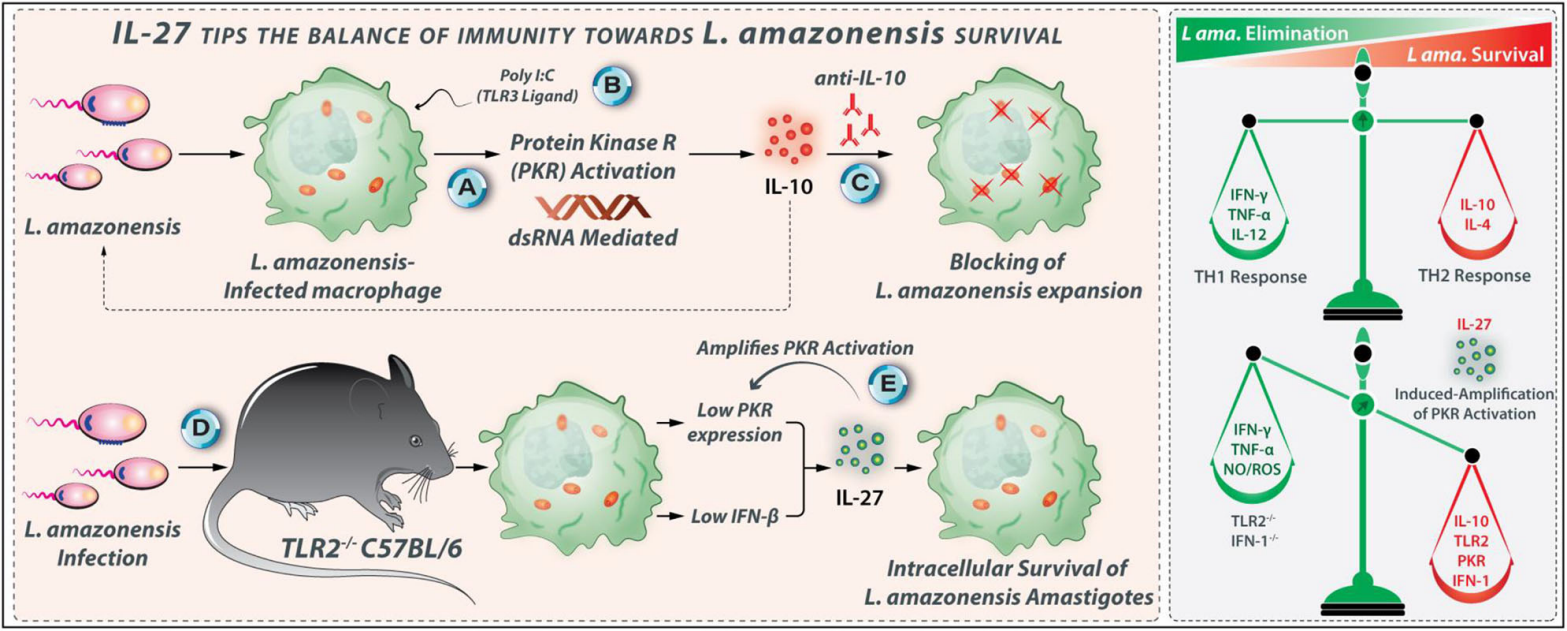
IL-27 tips the balance of immunity toward *L. amazonensis* survival. IL-27-related enhancing effects on *L. amazonensis* growth and survival occur mainly through IL-10. **(A)** Inside macrophages, *L. amazonensis* activates protein kinase R (PKR). **(B)** PKR activation can also occur through Poly I:C (a ligand for TLR3), which elicits IL-10 production. **(C)** Using anti-IL-10 antibody parasite replication could be entirely abrogated. **(D)** TLR2^−/−^ macrophages express low levels of IFN-β and PKR post-*L. amazonensis* infection, indicating that the TLR2-PKR-IFN-1 axis contributes to the IL-27 production during *L. amazonensis* infection that supports parasite survival. **(E)** Interestingly, when produced, IL-27 triggers more PKR activation, which triggers its production by a positive feedback mechanism.

It is reported that the addition of rIL-27 to human macrophages infected with *L. amazonensis* promastigotes augments the parasite propagation and percentage of infected cells measured on days 3 and 4 after the cytokine addition. The IL-27 neutralization or IL-10 receptor blockade in the infected cell cultures markedly reduce the parasite multiplication (116). Hence, the IL-27-related enhancing effects on *L. amazonensis* survival and growth occur mainly through IL-10. Treatment of the *L. amazonensis-*infected human macrophages with a PKR inhibitor abrogates the IL-27-mediated *L. amazonensis* replication. PKR is activated by IL-27, which is crucial to IL-27-mediated *Leishmania* expansion (116). TLR2-deficient C57BL/6 mice are also less susceptible to *L. amazonensis* infection than wild-type counterparts (63). TLR2^−/−^ macrophages express low quantities of IFN-β and PKR post*-L. amazonensis* infection (63). Indeed, *L. amazonensis* infection leads to the activation of the PKR/IFN-1 axis *via* a TLR2-dependent manner, which promotes parasite replication in macrophages (116). Interestingly, the *Leishmania*-induced IL-27 expression also depends on the TLR2 and IFN-1-mediated signaling (119). The expression of IL-27 is profoundly diminished in infected-macrophages derived from TLR2^−/−^ or IFN-1R^−/−^ mice (116, 119). TLR2-PKR-IFN-1 signaling contributes to the IL-27 production in *L. amazonensis-*infected macrophages that lead to the intracellular survival of the parasite. When produced, IL-27 elicits new cycles of PKR activation, which supports parasite expansion (116). These regulatory effects of IL-27 during *L. amazonensis* infection are depicted in Figure 7.

The *L. amazonensis*-infected macrophages may be one of the main sources of IL-27 during the infection. IL-27 profoundly increases the vulnerability *L. amazonensis* infection, and its promoting effects on the parasite survival and growth occur mainly through an IL-10-dependent manner. Indeed, the TLR2-PKR-IFN-1 axis contributes to the IL-27 production during *L. amazonensis* infection that supports the survival of the parasite. When produced, IL-27 triggers more PKR activation, which stimulates its production by a positive feedback mechanism. Accordingly, the targeting of TLR2, PKR, and IFN-1 may impair the IL-27 production, which eventually diminishes the *L. amazonensis* replication.

### Role of IL-27 in *L. braziliensis* Infection

**Figure 8:**
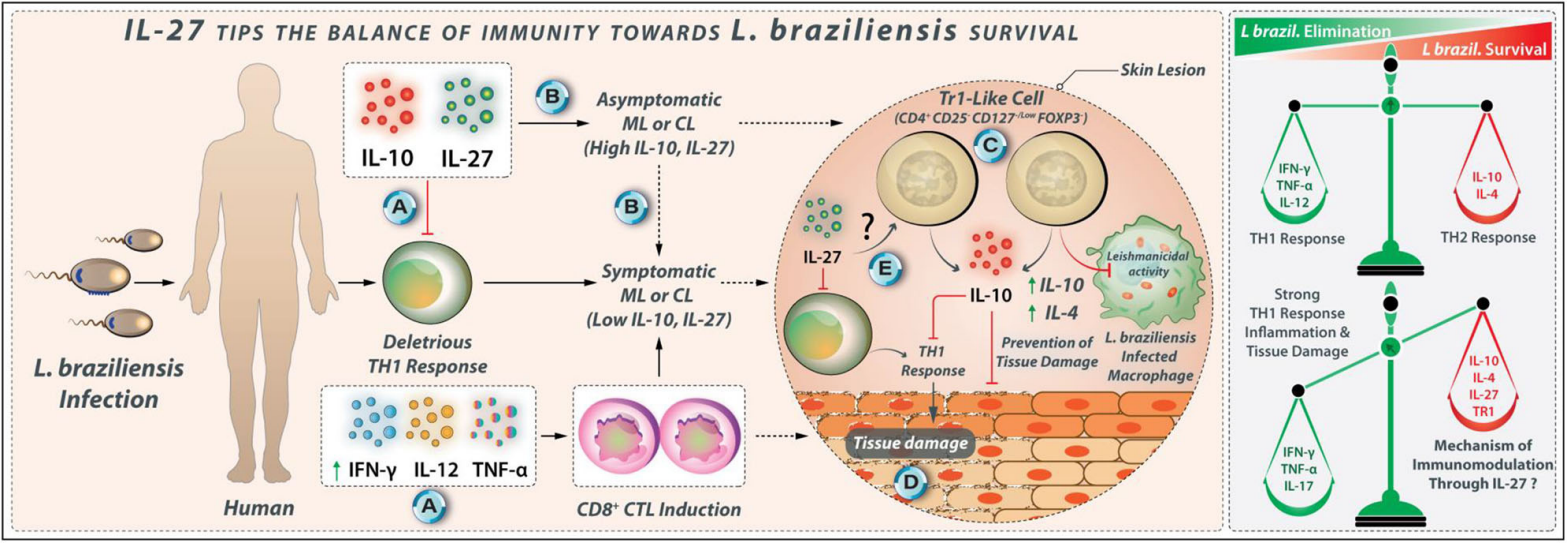
IL-27 tips the balance of immunity toward *L. braziliensis* survival. **(A)** IL-10 and IL-27 may modulate deleterious Th1 responses in *L. braziliensis* infection. **(B)** The proper expression of IL-10 and IL-27 may cause asymptomatic subclinical pattern, while the reduced expression of IL-10 and IL-27 may lead to symptomatic CL pattern in *L. braziliensis* infected individuals. **(C)** Tr1 cells with phenotype CD4^+^ CD25^−^ CD127^−/low^ FoxP3^−^ are identified in the peripheral blood of *L. braziliensis-*infected patients with CL that produce higher amounts of IL-10. **(D)** Tr1 cells at the initial phase of infection may suppress the Leishmanicidal mechanisms of macrophages. However, during the late stages of infection, IL-27, along with IL-10, attenuates tissue damage in *L. braziliensis* infection. **(E)** The mechanism through which Tr1 cells are induced by IL-27 is unknown to date.

The *L. braziliensis*-related CL is associated with robust Th1 responses (65, 120) conferring protection against the parasite. However, excessive uncontrolled inflammation is responsible for the development of skin lesions and tissue damages, as observed in ML and CL patients (118). Reduced IL-10 production along with the heightened IFN-γ and TNF-α production are positively correlated with lesions sizes and tissue damage in CL and ML (118, 121). A high number of cytotoxic cells are also observed in lesions of patients with CL caused by *L. braziliensis* and correlated with explicit inflammatory stress and cutaneous tissue damage (122). Patients treated with anti-inflammatory drugs show alleviated lesions size, supporting the deleterious role of inflammation during the *L. braziliensis* infection (65). The PBMCs from *L. braziliensis*-infected individuals with CL pattern produce higher levels of IFN-γ and TNF-α than cells from patients with subclinical (SC) pattern after *in-vitro* stimulation with SLA (123). The SLA-stimulated PBMCs from patients with SC tend to express higher amounts of IL-17 compared with patients with CL pattern (124). The addition of rIL-27 to SLA-stimulated PBMCs from patients with CL reduces IFN-γ production (81, 124). Further, higher IL-10 levels were detected in *L. braziliensis*-infected individuals with SC pattern when compared with CL patients (125). Therefore, IL-10 and IL-27 may modulate the deleterious Th1 cell responses in *L. braziliensis* infection. Hence, the proper expression of the IL-10 and IL-27 may cause an asymptomatic SC form, while the low expression of IL-10 and IL-27 may lead to symptomatic CL pattern in *braziliensis*-infected individuals (Figure 8).

#### IL-27 in *L. braziliensis* Infection

IL-10 and TGF-β, but not IL-27, modulate the expression of the TNF-α, IFN-γ, and IL-17 by SLA-stimulated PBMCs from ML and CL patients (118). IL-27 was unable to enhance IL-10 production by PBMCs from CL and ML patients (81). The PBMCs from CL and ML patients are unresponsive to the immunomodulatory effects of IL-27. In skin biopsy from patients with *L. braziliensis*-related CL, the increased IL-10 and IL-27 expression were also associated with high IFN-γ expression, although a similar pattern of cytokine expression was not observed in ML group (126). A positive association has been indicated between the IL-10 expression and IL-27, IL-21, and IFN-γ expression but not with the FOXP3 expression in lesions of patients with CL (127). A new subgroup of Treg cells, namely Tr1-like cells with CD4^+^ CD25^−^ CD127^−/low^ FOXP3^−^ phenotype was identified in the peripheral blood of *L. braziliensis*-infected patients with CL that produce higher levels of IL-10 (127). The secretion of IL-10 from CD4^+^ CD25^−^ FOXP3^−^ cells in the initial phase may suppress the Leishmanicidal mechanisms of macrophages, which supports the establishment of infection (127). However, in chronic stages, when a strong Th1 response is dominant, Tr1-like cells may help in protecting tissues from damage caused by an inflammatory reaction. The possible IL-27 contribution to the development of the Tr1-like cells needs to be further investigated. The regulatory effects of IL-27 during *L. braziliensis* infection are shown in Figure 8.

Collectively, the excessive uncontrolled Th1 cell-mediated inflammation is involved in the tissue damages in ML and CL *L. braziliensis*. The IL-27, in parallel with IL-10, attenuates the deleterious Th1 cell response during the late stage of the *L. braziliensis* infection (124). However, the precise role of the IL-27 during the early stage of *L. braziliensis* infection needs more clarification.

## Conclusion

IL-27 is expressed during the early phase of *Leishmania* infection, and the infected phagocytes and DCs are the major producers of this cytokine. The role of IL-27 in *Leishmania* infection is species-dependent (Table 1). The protective role of IL-27 during the initial stage of *L. major* infection is exerted mainly through inhibition of IL-4-mediated Th2 cell responses. Therefore, evaluation of the local therapeutic potential of IL-27 or its agonists concerning the *L. major*-mediated CL requires more investigations. In mouse models of OVA-induced asthma, the intranasal IL-27 administration significantly improves the clinical symptoms of the disease, decreases the local eosinophilia in the nasal mucosa, modulates the cytokine production by Th1, Th2, and Treg cells, and decrease the serum levels of specific IgE (128, 129). The effects of such intranasal IL-27 administration need to be investigated in various *Leishmania sp*. infections.

**Table 1:** Experimental studies indicating the role of IL-27 in Leishmaniasis disease.

**Parasite species**	**Mouse strain**	Infective dose^*^	**Infection site**	**Observation**	**References**
*L. major*	WT C57BL/6	1 × 10^3^ MP	Ear dermis	• P28 expression is increased in infected tissue at week two post-infection. • EBI3 expression becomes detectable in infected tissue at week five post-infection.	(68)
*L. major*	WSX-1^−/−^ C57BL/6	1 × 10^3^ MP	Ear dermis	• The mice exhibit more severe lesions that were associated with the appearance of the IL-17^+^ CD4^+^ cells. • IL-27 administration prevents the development of abnormal Th17 cells during Leishmaniasis. • The mice display a low percentage of IL-10^+^ IFN-γ^−^ CD4^+^ cells and more severe lesions at week 6 post-infection.	(68)
*L. major*	WT C57BL/6	2 × 10^5^ MP	Ear dermis	• CD11c^+^ DCs express P28 and EBI3 in draining lymph nodes at 24 h post-infection.	(69)
*L. major*	EBI3^−/−^C57BL/6	2 × 10^5^ MP	Ear dermis	• Mice display greater susceptibility to infections with maximum parasites loads and lesion sizes at week six post-infection. • At weeks 2 and 4 post-infection, the IFN-γ secretion is lower, while the IL-4, IL-10, and IL-13 secretion were higher by *L. major* lysate-stimulated lymphocytes. • At early time points after infection, the Th1/Th2 balance is diverted toward Th2 cells. • Protective immunologic memory is not impaired in EBI3^−/−^ mice. • IL-27 is essential for early control of parasite replication.	(69)
*L. major*	WSX-1^−/−^ C57BL/6	2 × 10^6^ MP	Hind footpad	• Lymph node cells produce low *Leishmania*-induced IFN-γ levels at the initiation of infection. • Lymph node cells produce normal IFN-γ levels later in infection. • Mice produce considerable levels of IL-4 at the initial of infection. • Susceptibility to *L. major* is limited to the initial stages of infection. • The IP administration of anti-IL-4 antibody (every 4 days for the first 4 weeks of after infection) improves the IFN-γ production and host resistance. • The treatment with blocking anti-IL-4 antibody increases the number of IL-17^+^ CD4^+^ cells.	(71)
*L. major*	WT BALB/c	2 × 10^5^ PM	^†^S.C into the hind footpad	• Administration of IL-27 reduces the parasite load. • Administration of IL-27 increases the survival rate. • Lymph node cells from IL-27-treated *mice* secrete higher IFN-γ quantities and lower IL-4 levels.	(34)
*L. donovani*	WT C57BL/6	3 × 10^7^ AM	Intravenously	• Splenic CD11c^hi^MHCII^hi^ cDCs collected at days 21 and 28 post-infection express high IL-27 levels. • The DC-derived IL-27 may enhance the IFN-γ^+^ IL-10^+^ CD4^+^ cell polarization *in vivo*.	(94)
*L. donovani*	WT BALB/c	2 × 10^7−8^ AM	Intravenously	• Infection promotes the IL-27 expression by splenic CD8α^+^ and CD4^+^ DC at days 1, 14, and 28 post-infection.	(90)
*L. donovani*	WSX-1^−/−^ C57BL/6	1 × 10^7^ AM	Intravenously	• Mice contain fewer parasites in livers on days 15, 30, and 60 after infection. • Mice show high resistance to *L. donovani* infection. • Mice display higher serum IFN-γ and TNF-α level on day 15 post-infection. • Mice display higher serum IL-12 levels on day 30 post-infection. • By day 60, levels of IL-12, TNF-α, and IFN-γ drop in mice. • The *Leishmania* antigen-stimulated spleen cells produce more amounts of IFN-γ and IL-12 on day 30 post-infection and produce more significant levels of NO on days 15 and 30 after infection. • Mice mount a robust Th1 cell response after *L. donovani* infection. • Mice display severe liver pathology. • The depletion of CD4^+^ T cells reduces liver pathology. • Neutralization of both TNF-α and IFN-γ reduces liver pathology.	(92)
*L. donovani*	WT BALB/c	2.5 × 10^7^ AM	Intravenously	• Administration of the neutralizing anti-EBI3 antibody (and not anti-p28 antibody) to pathogen-infected mice reduces the parasite load in the spleen and liver and increase the TNF-α- and IFN-γ-secreting cells.	(101)
*L. infantum*	WT C57BL/6 WT BALB/c	1 × 10^8^ PM	Intravenously	• Serum IL-27 levels are increased early (at 4 days after infection) in the BALB/c mice, but not in C57BL/6 mice. •l The splenic DCs from BALB/c mice but not from C57BL/6 mice upregulate the expression of IL-27p28 24 h after infection. • IL-27 secretion by BMDCs from BALB/c was higher compared to C57BL/6, whereas the LPS-stimulated BMDCs from C57BL/6 mice produce more elevated amounts of IL-27 than BALB/c mice. • The administration of rIL-27 to in C57BL/6 increase the production of IL-10, while decrease IFN-γ and IL-12p70, and prevent the infiltration of neutrophils in the spleen at 24 h after treatment in comparison with infected non-treated animals. • Neutralization of IL-27 in acutely infected BALB/c decreases parasite burdens transiently reduces IL-10 and a transient increase in splenic IFN-γ producing CD4^+^ and CD8^+^ T cells.	(102)
*L. infantum*	WT C57BL/6	1 × 10^7^ PM	Intravenously	• High levels of the P28 subunit of IL-27 are detected in the spleen and liver at weeks 4 and 6 post-infection. • The expression of both IL-27R subunits, including WSX-1 and gp130, are upregulated at week 4 after infection.	(103)
*L. infantum*	EBI3^−/−^ C57BL/6	1 × 10^7^ PM	Intravenously	• The parasite loads in the liver and spleen were lower at weeks 4 and 6 post-infection. • Mice produce higher IL-17A levels in both spleen and liver. • Restimulation splenocyte with *L. infantum* lysate leads to more IL-17A production. • Higher CXCL1expression was observed in the spleen, which causes a peak in neutrophil migration at week 4 post-infection in the spleen and liver. • When IL-17 was blocked, the mice become as susceptible as the C57BL/6 control.	(103)
*L. amazonensis*	WT C57BL/6	5 × 10^5^ PM	Hind footpad	• The IL-27 administration into the infected footpads (on days 2, 4, and 6 after infection) enhances the lesion sizes and parasite number in the footpads and the draining lymph nodes in weeks 2 and 3 following infection.	(116)

* AM, Amastigotes; MP, Metacyclic promastigotes; PM, Promastigotes;

† *SC, Subcutaneous*.

*Leishmania* skews naive T cells toward Th2 cells for its survival in the host. On the other hand, IL-27 has the capability to polarize naïve T cells to Th1 cells, which plays host protective roles by jeopardizing parasite growth in the host. Nonetheless, IL-27 may suppress the protective immune responses against *L. donovani, L. infantum, L. amazonensis*, and *L. braziliensis*. Therefore, T cell-dependent IL-27 functions are diverse, species-specific, and contradictory. At the same time, the infection and pathology of the *Leishmania* species are unique and vary with IL-27 dependency. The targeting of IL-27 or its receptor using blocking monoclonal antibodies, small molecule inhibitors, and siRNA, alone or in combination with other therapeutic agents, needs to be evaluated as promising strategies for the treatment of the *L. donovani-, L. infantum-, L. amazonensis-, and L. braziliensis-*related complications. IL-27 or IL-27 associated signaling molecules may be considered as exciting novel targets for immunomodulation in Leishmaniasis. Further, the clinical values of IL-27 as a marker of Leishmaniasis severity and/or as a monitoring marker of treatment efficacy need more considerations. It may be underlined that IL-27 possesses the potential to be a valuable biomarker for active human VL and for monitoring the effectiveness of treatment modality.

## Author Contributions

AJ, MN, and IS conceptualized and drafted the manuscript and Table 1. BS and AS scrutinized the scientific-content, language, grammar, and edited the manuscript. AP and PC contributed additionally to writing and reviewed the manuscript. PC conceptualized, designed, and digitalized the figures. All authors contributed to the article and approved the submitted version.

## Conflict of Interest

The authors declare that the research was conducted in the absence of any commercial or financial relationships that could be construed as a potential conflict of interest.

## Abbreviations

AhR: aryl hydrocarbon receptor
AP1: activator protein 1
APC: antigen presenting cell
CCR: C-C chemokine receptor
CL: cutaneous Leishmaniasis
CTLA-4: cytotoxic T-lymphocyte–associated antigen 4
CXCL: chemokine (C–X–C motif) ligand
DC: dendritic cell
DCL: diffuse cutaneous Leishmaniasis
EBI3: Epstein-Barr virus-induced gene 3 (EBI3) subunits
ERK: extracellular signal-regulated
GAS: gamma interferon activation site
IDO: inoleamine-2,3-dioxygenase
IFN: interferon
IKK: I kappa B kinase
IL: interleukin
ILCs: innate lymphoid cells
iNOS: inducible nitric oxide synthase
IRAK: interleukin-1 receptor-associated kinase 4
IRF: interferon regulatory factor
ISRE: interferon-sensitive response element
JAK/STAT: Janus kinase/signal; transducers and activators of transcription
JNK: Janus kinase
LPS: lipopolysaccharide
LRR: leucine-rich repeats
MAL: MyD88 adaptor-like protein
MAPK: mitogen-activated protein kinases
MCL: mucocutaneous Leishmaniasis
MHC: major histocompatibility complex
MIP: macrophage inflammatory proteins
MKK: mitogen-activated protein kinase
moDCs: monocyte-derived dendritic cells
MyD88: MYD88 innate immune signal transduction adaptor
NAP-1: nucleosome assembly protein
NET: neutrophil extracellular traps
NF-κB: nuclear factor Kappa-light-chain-enhancer of activated B cells
NK cells: natural killer cells
NKT: natural killer T cells
NO: nitric oxide
NOX: nicotinamide adenine dinucleotide phosphate (NADPH) oxidase
p38MAPK: P38 mitogen-activated protein kinases
PAMPs: pathogen-associated molecular patterns
PD-1: programmed cell death protein 1
PD-L2: programmed cell death ligand 2
PI3K: phosphoinositide 3-kinase
PKDL: post-kala-azar-dermal Leishmaniasis
PKDL: Post-kala-azar dermal Leishmaniasis
PKR: protein kinase R
PRR: pattern recognition receptors
RNI: reactive nitrogen intermediates
RORγT: RAR-related orphan receptor gamma
ROS: reactive oxygen species
SOCS: suppressor of cytokine signaling
STAT: signal transducer and activator of transcription
TAB: TAK1 binding protein
TAK1: transforming growth factor beta-activated kinase 1
T-Bet: T-box transcription factor
TBK-1: tank binding kinase-1
TCCR: T-cell cytokine receptor
TGF: transforming growth factor beta
TLR: toll-like receptor
TNF-α: tumor necrosis factor alpha
TRAM: TRIF-related adaptor molecule
TRIF: TIR-domain-containing adapter-inducing interferon-β
VL: visceral Leishmaniasis.
